# Assessment of the Fascial System Thickness in Patients with and Without Low Back Pain: A Narrative Review

**DOI:** 10.3390/diagnostics15162059

**Published:** 2025-08-16

**Authors:** Lorenza Bonaldi, Alice Berardo, Antonio Stecco, Carla Stecco, Chiara Giulia Fontanella

**Affiliations:** 1 Department of Civil, Environmental and Architectural Engineering, University of Padova, 35131 Padova, Italy; lorenza.bonaldi@phd.unipd.it; 2 Centre for Mechanics of Biological Materials, University of Padova, 35131 Padova, Italy; carla.stecco@unipd.it (C.S.); chiaragiulia.fontanella@unipd.it (C.G.F.); 3 Department of Industrial Engineering, University of Padova, 35131 Padova, Italy; 4 Department of Rehabilitation Medicine, NYU Grossman School of Medicine, New York City, NY 10016, USA; antonio.stecco@gmail.com; 5 Department of Neuroscience, Institute of Human Anatomy, University of Padova, 35121 Padova, Italy

**Keywords:** low back pain, thickness, superficial fascia, deep fascia, subcutaneous tissue

## Abstract

**Background and Objectives:** The hypothesis that fascial thickness variability may serve as a biomarker for low back pain (LBP) requires a clear understanding of typical thickness values in both LBP and non-LBP populations—an area still lacking in the literature. This narrative review aims to define reference values and patterns of variability for the superficial fascia, deep fascia, and subcutaneous tissue in individuals with and without LBP. **Methods:** A literature search was conducted in PubMed and ScienceDirect using keywords such as *superficial fascia*, *deep fascia*, *thoracolumbar*, *subcutaneous fat*, *back pain*, *lumbar*, *thorax*, and *thickness*. Inclusion criteria focused on human studies with proper identification of the relevant soft tissue structures. A total of 21 studies, published up to February 2024, met the inclusion criteria and were analyzed. **Results:** The review revealed notable intra- and inter-study variability in the thickness of the investigated structures. In LBP populations, both deep fascia and subcutaneous tissues were generally equal to or thicker than in controls (non-LBP), whereas consistent data on superficial fascia thickness remain limited. Age, sex, and anatomical location were discussed as potential influencing factors. **Conclusions:** These findings support the establishment of reference thickness values for subcutaneous and fascial tissues and encourage further investigation into their structural and functional roles in LBP. The observed variability may offer a basis for patient- and site-specific assessment and intervention strategies.

## 1. Introduction

The fascial tissues are part of a system that is a multilayered structure composed of loose (adipose cells, glycosaminoglycans, and hyaluronan) and dense connective tissues (collagen fibers). From the skin to the deepest plane, it comprises the superficial fascia (SF) and the deep fascia (DF) [1,2]. The SF and DF are connected through the superficial and deep retinacula cutis, respectively, which organize the adipose tissue into lobules. Both these fascial tissues have different compositions, structures, and mechanical properties, which vary further depending on the body region. In the thoracolumbar region, the SF appears as a fibroadipose structure composed of interconnected lamellae that lie between the dermis and the DF. The DF, also known as the thoracolumbar fascia (TLF), is an aponeurotic fascia consisting of multiple sublayers. The SF and DF often merge near the spinous processes. The SF and DF are frequently assumed to share similar anatomical and physiological properties, leading to misleading clinical interpretations. This study aims to further investigate the distinct morphology of these two fascial tissues. In the last decades, the fascial tissue has gained increasing interest due to its role in musculoskeletal dysfunctions, such as low back pain (LBP) [3,4,5]. LBP is still the leading cause of disability in the world [6,7], and the importance of investigating the fascia is because fascial tissue, such as the TLF, is assumed to have a direct and significant implication in LBP, even for its treatment [3,4,5]. Indeed, TLF mobility seems to decrease in people with LBP [8]. Quantitatively speaking, Langevin et al. [5] demonstrated that TLF shear strain (i.e., gliding among connective tissue planes) is reduced by approximately 20% in human subjects with LBP. Anatomically, the above-mentioned TLF is a trilaminar structure that connects the trunk with the upper/lower limbs and that separates the paraspinal muscles from the posterior abdominal wall muscles. Indeed, the superficial lamina of the posterior layer is dominated by the aponeuroses of the latissimus dorsi and the serratus posterior inferior, while the deeper lamina encapsulates the paraspinal muscles. Moreover, the TLF is characterized by a nociceptive nature, as evidenced by its sensitivity to stimuli [9,10,11,12,13,14,15]. For example, Schilder et al. [16] showed that the TLF is sensitive to chemical stimulation, making it prone to contribute to non-specific LBP. Additionally, the biomechanics of the TLF are strongly influenced by its direct connection with muscles [15], thus increasing its spectrum as a potential source of pain. According to Creze et al. [17], the TLF, as well as the erector spinae aponeurosis, is fundamental in the biomechanics of the spine and could be a root cause of LBP.

Therefore, both anatomical and functional alterations could have a fundamental impact. In these terms, Brandl et al. [18] affirmed that alterations in posture, kinematics, and movement patterns are commonly associated with LBP patients, and that TLF has a fundamental impact on spinal stability and paraspinal muscle activity. For instance, the authors demonstrated that TLF deformation could be a suitable parameter to distinguish between LBP and healthy individuals during body movement, such as a lifting task. In another study, Brandl et al. [19] reported that in LBP participants, the erector spinae and multifidus muscles’ activity is significantly affected by TLF, and the hypothesized mechanism (as the root cause) behind it is fascia-related disturbances in neuromotor control due to altered muscle spindle functions. Moreover, Kellis et al. [20] demonstrated that TLF stiffness is remotely influenced by hamstring muscle movements. In addition, according to D’Hooge et al. [21], it is hypothesized that degenerated muscles might also compromise spinal stability and lead to further injury/pain. In terms of other factors that could influence LBP, such as fat, Heuch et al. [22] demonstrated that obesity is associated with a high prevalence of LBP. Other authors [23] have affirmed that BMI (as an indicator of obesity, which is a risk factor for LBP) cannot represent the percentage and distribution of body fat. Thus, subcutaneous fat tissue thickness has been demonstrated to be better than BMI in predicting a symptomatic patient with LBP (two times thicker compared to asymptomatic subjects at all lumbar levels). Additionally, Brooks et al. [24] examined the relationship of regional and total body adiposity to pain and disability in an LBP population, concluding that a regional distribution (score: ratio between abdominal and lumbar adiposity) is associated with LBP.

Even if the correlation between musculoskeletal dysfunctions, such as LBP, and fascial system substrate thickness variation (i.e., SF, DF, and subcutaneous tissue) is hypothesized [2], it is not clear whether it could be a consequence or the main cause of the bodily alterations.

Therefore, to evaluate the change in thickness between the LBP and non-LBP groups, a comprehensive evaluation and description of thickness variability within both groups is a key step for further clinical and treatment evaluations.

The hypothesis of a thickness variation in the LBP scenario, and according to variables such as sex, age, and BMI, requires the definition of thickness variability in non-LBP and LBP.

For all of the above-mentioned reasons, the aim of the present work was to comprehensively review the literature to describe and discuss the thicknesses of the fascial layers (i.e., SF and DF) and surrounding structures (i.e., local subcutaneous tissue thickness in the thoracolumbar region) to determine their variability in non-LBP and LBP scenarios.

Given the heterogeneity of the included studies (detailed in the next section) in terms of anatomical definitions, measurement protocols, and reported outcomes, a narrative review format was chosen. This approach allowed for a more comprehensive and integrative analysis of the available literature, with the flexibility to include relevant conceptual insights and observational findings that would be excluded under a more rigid systematic framework.

Comparing literature could be a useful tool for defining, as a future perspective, the implications in LBP. The main expected outcome of this work was to identify consistent anatomical differences or patterns of variability in fascial and subcutaneous tissue thickness associated with LBP. Such findings could have important implications in clinical practice, particularly for improving diagnostic imaging criteria, tailoring treatment strategies based on patient-specific fascia-related parameters, and informing future interventional studies focused on fascial therapy.

## 2. Materials and Methods

### 2.1. Research Questions

This review aimed to investigate the thickness variability of fascial tissues in the thoracolumbar region with or without low back pain (LBP vs. non-LBP) by addressing two specific research questions: (1) What are the typical thickness values and their variability for the superficial fascia (SF), deep fascia (DF), and subcutaneous tissues in the thoracolumbar region in asymptomatic individuals? (2) How do these thicknesses differ between individuals with and without LBP, also in association with other factors (e.g., sex, age, and surrounding structures)?

These questions were developed to support a comprehensive review of the literature and to guide a comparative analysis of fascial anatomy in symptomatic and asymptomatic LBP populations.

### 2.2. Keywords

To answer the identified research questions, the current narrative review examined the literature in the following databases: PubMed and ScienceDirect. The search was conducted before February 2024. Keywords present in the title and abstract included superficial fascia, deep fascia, thoracolumbar, subcutaneous fat, back pain, lumbar, thorax, and thickness. The queries were (i) “superficial fascia” and (“back pain” or trunk or lumbar or thorax) and thickness; (ii) (“deep fascia” or thoracolumbar) and (“back pain” or trunk or lumbar or thorax) and thickness; (iii) “subcutaneous fat” and (“back pain” or trunk or lumbar or thorax) and thickness. Additional articles were added from references or other sources, such as websites.

### 2.3. Article Inclusion Criteria

Inclusion criteria were (i) human studies (subjects > 18 years old), (ii) presence of quantitative information (e.g., thickness measurement values), and (iii) proper structure identification (i.e., SF). Exclusion criteria also pertained to articles published in languages other than English.

**Figure 1:**
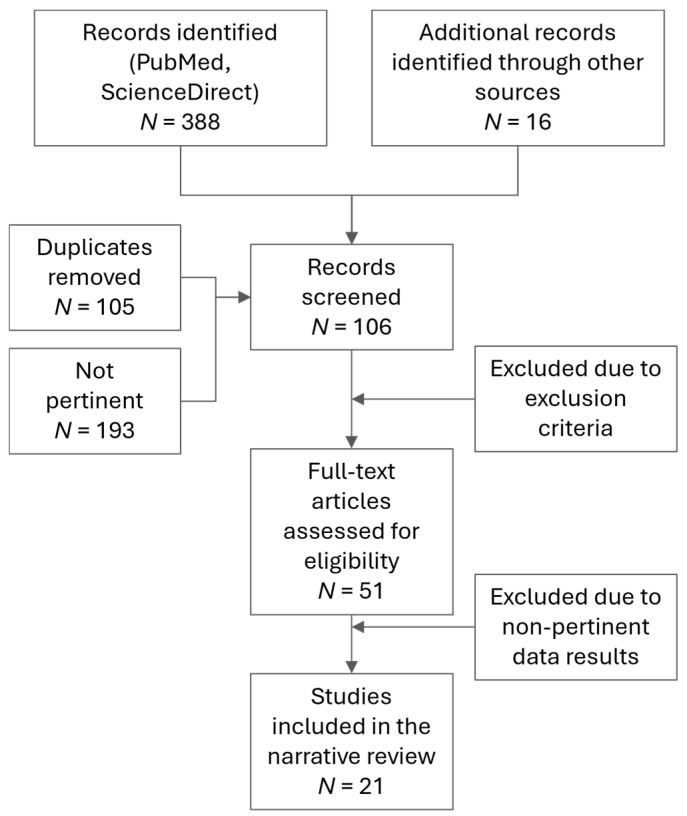
Flow diagram of the study selection process adapted for this narrative review. A total of 388 records were identified through database searches, and 16 through other sources. After removing 105 duplicates and 193 non-pertinent works, 106 records were screened by title and abstract. Of the 51 full-text articles assessed, 21 met the inclusion criteria and reported pertinent results regarding the research questions of this review; thus, they were included. Exclusion reasons included non-human studies, absence of quantitative data, or improper anatomical structure identification.

**Figure 2:**
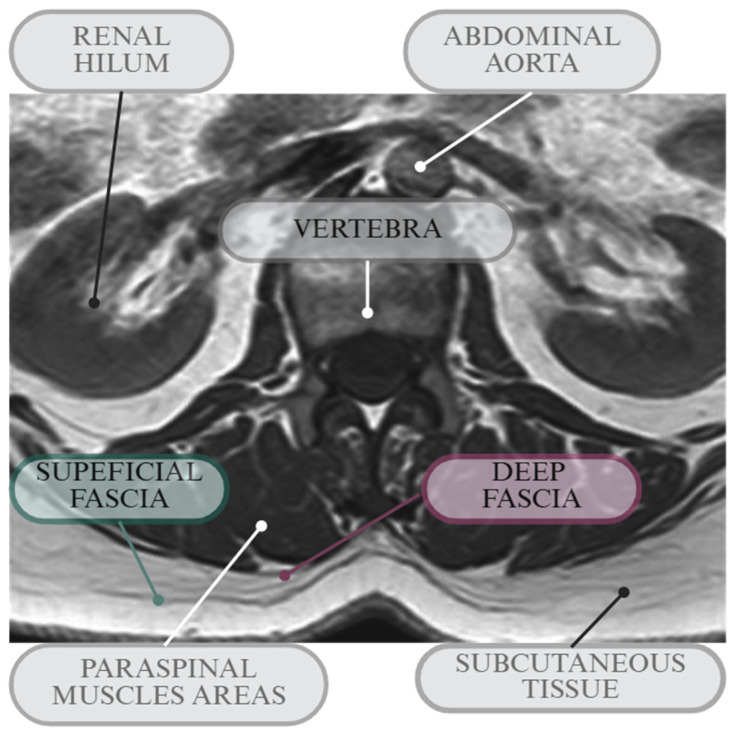
Example MRI (axial, T2-weighted) with the relative anatomical structures (created in BioRender. Bonaldi, L. (2025) https://BioRender.com/n2jgy4l).

The articles were first screened according to the title and abstract, and secondly, in full text by one of the authors. Since the review has been presented in a narrative form, the screening flowchart with the number of articles according to PRISMA guidelines [25] is not reported. However, for clarity, the rationale is provided, adapted from the PRISMA one (Figure 1). Finally, a total of 21 relevant works presenting clear numerical values were organized into the following table that summarized all the study characteristics (Table 1). An example of the anatomical structures of interest is shown in Figure 2. Other works that presented interesting observations about the topic were included in the text for discussion. Three other experts in the field verified the validity of the analysis and added additional considerations to the review.

## 3. Results

The articles included in the analysis are reported in Table 1 for comparison purposes. A detailed breakdown of fascial thickness values is presented in Table 2, Table 3 and Table 4. For clarity, key trends are briefly summarized here, while full data are retained in the tables for transparency and future reference.

An overview of the included studies is presented in Table 1, detailing sample size, sex distribution, BMI, age, spinal level examined, and measurement methodology. Among the 21 studies reviewed, 16 employed ultrasound (US) as the primary imaging modality, four were conducted ex vivo, and two used MRI, reflecting a predominant reliance on non-invasive imaging for in vivo assessments. Regarding anatomical focus, the DF was investigated in 19 studies, the SF in two studies, and subcutaneous tissue thickness in eight studies, with some works examining multiple structures concurrently (84% for DF, 50% for SF, and 63% for subcutaneous tissue). The spinal levels most frequently assessed were L2–L5, although a few studies extended the analysis to thoracic regions or the full lumbar spine. Study populations varied widely, including both LBP and non-LBP individuals, with diverse age ranges (from young adults to elderly participants) and BMIs. Sex distribution was mixed in most studies, though a few included only males or females. Detailed characteristics are summarized in Table 1.

**Table 1:** Study characteristics of the analyzed cohort extracted from the Materials and Methods section of the reported references. Non-LBP groups include subjects without specific evidence of LBP. US: ultrasound; M: male; F: female; BMI: body mass index; SF: superficial fascia; DF; deep fascia; LBP: low back pain s.d.: standard deviation.

Structure	Reference	Study Population (Number)	Sex	BMI (kg/m^2^)	Age (Mean ± s.d.)	Spinal Level	Method
**SF**	[26]	10	M 100%	23.06 ± 2.6	30.6 ± 4.99 (non-LBP)	Posterior chest	US
	[27]	6	M 50%, F 50%	-	73–85 (not specified)	-	Ex vivo
**DF**	[28]	18	F 100%	23 ± 4	22 ± 1 (non-LBP)	L2	US
		17		27 ± 4	69 ± 4 (non-LBP)		
	[4]	107 (47 non-LBP, 60 LBP)	M (43% non-LBP, 42% LBP) F (57% non-LBP, 58% LBP)	25.9 ± 0.7 (non-LBP) 25.7 ± 0.6 (LBP)	39.3 ± 14.1 (non-LBP) 38.3 ± 13.3 (LBP	L2–L3	US
	[5]	121 (50 non-LBP, 71 LBP)	M (24 non-LBP, 38 LBP) F (26 non-LBP, 33 LBP)	26.1 ± 0.6 (non-LBP) 26 ± 0.5 (LBP)	41.8 ± 2.3 (non-LBP) 44.6 ± 1.8 (LBP)	L2–L3	US
	[29]	48	M 50%, F 50%	24.5	37.4 ± 13.3 (LBP)	L2–L3	US
	[30]	22	-	Lower than 28.5 (exclusion criteria)	25–65 (LBP)	L2–L3	US
	[31]	66	M 100%	23 ± 1.5	22.6 ± 3.7 (non-LBP)	L2–L3	US
	[32]	50	M 58%, F 42%	23.91 ± 3.58	36 (non-LBP)	L3	US
	[33]	92	M 49% F 51%	24.03 ± 6.1 (non-LBP) 23.37 ± 5.22 (LBP)	27.09 ± 12.38 (non-LBP) 28.96 ± 10.54 (LBP)	L3	US
	[20]	14	M 100%	Mass 78.9 ± 8.02 kg Height 181 ± 9.71 cm	23.7 ± 7.31 (non-LBP)	L3–L4	US
	[34]	54	M (12 non-LBP, 10 LBP) F (19 non-LBP, 13 LBP)	22.30 ± 2.00 (non-LBP) 22.11 ± 2.84 (LBP)	22.94 ± 5.23 (non-LBP) 25.13 ± 10.04 (LBP)	L4	US
	[35]	30	M 100%	24.03 ± 2.14	24 ± 5 (non-LBP) 28 ± 10 (LBP)	L4	US
	[36]	63	M (15 non-LBP, 15 LBP) F (15 non-LBP, 18 LBP)	24.4 ± 3.2 M (non-LBP) 23.3 ± 3.6 (F non-LBP) 25.8 ± 3.9 M (LBP) 26.8 ± 3.0 (F LBP)	Non-LBP: 39.3 ± 14.3 (M) 39.8 ± 14.1 (F) LBP: 44.5 ± 13.9 (M) 47.8 ± 11.9 (F)	L4–L5	US
	[37]	65	M (15 non-LBP, 16 LBP) F (15 non-LBP, 19 LBP)	24.4 ± 3.2 M (non-LBP) 23.3 ± 3.6 (F non-LBP) 25.6 ± 3.8 M (LBP) 26.8 ± 3.0 (F LBP)	Non-LBP: 39.3 ± 14.3 (M) 39.8 ± 13.7 (F) LBP: 44.1 ± 13.5 (M) 47.8 ± 11.9 (F)	L4–L5	US
	[38]	63	M (15 non-LBP, 15 LBP) F (15 non-LBP, 18 LBP)	24.4 ± 3.2 M (non-LBP) 23.3 ± 3.6 (F non-LBP) 25.8 ± 3.9 M (LBP) 26.8 ± 3.0 (F LBP)	Non-LBP: 39.3 ± 14.3 (M) 39.8 ± 14.1 (F) LBP: 44.5 ± 13.9 (M) 47.8 ± 11.9 (F)	L4–L5	US
	[11]	20	-	-	50–75 (not specified)	At different level	Ex vivo
	[26]	Reported above					
	[39]	29	F 100%	20.4 and 22.4 according to different testing groups	22.5 (18–29) (not specified)	n/a	US
	[17]	10	M 40% F 60%	-	77 ± 10 (non-LBP)	n/a	Ex vivo
	[40]	40	M 45% F 55%	-	59–84 (non-LBP)	n/a	Ex vivo
**Subcutaneous tissue**	[4]	Reported above					
	[30]	Reported above					
	[32]	Reported above					
	[37]	Reported above					
	[36]	Reported above					
	[41]	165	M 47% F 53%	-	39.1 (LBP)	L3–S1	MRI
	[23]	280	M (50 non-LBP, 52 LBP) F (50 non-LBP, 111 LBP)	24.5 ± 2.7 (non-LBP) 26.7 ± 4.3 (LBP)	32.5 ± 8.1 (non-LBP) 35.8 ± 6.8 (LBP)	L1–S1	MRI
	[26]	Reported above					

### 3.1. Deep Fascia Thickness

The DF of the lumbar region is also called the TLF, an aponeurotic fascia made up of multiple sublayers [2]. In the literature, different authors have reported TLF thickness (as a composite or single layer) in LBP and/or non-LBP populations (e.g., from in vivo ultrasound imaging or ex vivo evaluations; Table 2). From the literature analysis, in general, thickness values exhibited a significant variability; for example, those from the LBP group were equal to or higher (not always with statistical significance [5,33]) than the non-LBP ones. In detail, refs. [5,33] reported a significantly higher thickness in LBP subjects with respect to non-LBP ones (even if for the latter only for males). In any case, these authors assessed thickness at different levels and from populations of different ages (even if with potential confounding effects).

Langevin et al. [4] showed a 25% increase in perimuscular thickness in LBP subjects, without a specific correlation of thickness with age, but with BMI. Larivière et al. [38] analyzed the thickness of the posterior layer of the TLF in both LBP and non-LBP populations, also without finding a correlation with demographic data (age and sex). Meanwhile, Wilke et al. [28] investigated only non-LBP subjects reporting higher TLF thickness with increasing age. Loukas et al. [40] investigated the anatomy of the vertebral aponeurosis part of the posterior layer of the TLF (from non-LBP subjects), again without significant variations between sex, age, or body sides. Devantery et al. [29] discussed the perimuscular zone thickness of the TLF in subjects with LBP, also dividing it by body side, but focusing before and after treatment (i.e., myofascial technique). These authors demonstrated that the myofascial technique showed a significant decrease in TLF thickness in subjects with LBP, discussing that the decrement in thickness after treatment could be due to a change in hyaluronan viscosity (e.g., [42]), fluid movement, retinacula cutis, and the autonomic system [29]. To validate the claim that treatments affect tissue thickness in LBP subjects, Sanjana et al. [30] investigated the effect of hands-off, self-treatment (MELT) in reducing connective tissue thickness (thickness calculation based on Langevin et al. [4]). Meanwhile, Yang et al. [31] reported that percussive massage therapy does not change TLF thickness, even if referring to non-LBP male individuals (including the epimysial fascia in the calculation). Venkatesan et al. [43] reported that the effect of yoga on TLF thickness remains unclear, so there is still room for further investigations. On the variation of thickness along body planes, Marpalli et al. [11] measured the thickness of the posterior layer of the fascial tissue in formalin-embalmed human cadavers at different spinal levels, demonstrating that the thickness shows a cranio-caudal increase (but without distinction between LBP and non-LBP). Meanwhile, Creze et al. [17] evaluated the thickness of both the posterior (higher thickness) and middle layers of the TLF from fresh cadavers of a non-LBP population. In contrast, in non-LBP subjects, Hammoudeh et al. [26] compared the DF of different body regions, demonstrating greater thickness in the posterior part of the thorax, followed by the lumbar region. In fact, Hammoudeh et al. [26] investigated not only the DF but also the superficial one at different body sites, concluding that both are thick in the so-called high-tension areas, i.e., the upper trunk.

**Table 2:** Thickness (mean ± standard deviation) of the DF from the literature—divided between LBP and non-LBP subgroups. Unique cell: not specified or not considered separately between LBP and non-LBP subjects. Gray cells: works that include LBP and non-LBP groups for comparisons. * Mean ± standard error. Standard deviations and subgroup details are reported as presented in the original articles. In cases where such information was not available, missing values in our tables reflect the absence of data in the primary sources.

Reference	Thickness (mm)	
	Non-LBP	LBP
[28]	**1.33**	-
	**2.35**	
[4]	-	**+25%**
[5] *	**3.70 ± 0.40 (M)** **4.10 ± 0.30 (F)**	**4.90 ± 0.30 (M)** **4.10 ± 0.30 (F)**
[29]	-	Before treatment (divided into two populations) **2.00 ± 0.64 and 1.80 ± 0.45 (right)** **1.90 ± 0.50 and 1.78 ± 0.42 (left)**
[30]	-	**0.36 (left)** **0.34 (right)**
[31]	**2.56 ± 0.69 (left)** **2.69 ± 0.67 (right)**	-
[32]	**2.8** (median value and referred to by authors as SF)	-
[33]	**1.75 ± 0.85**	**2.11 ± 0.65**
[20]	**<3.00**	-
[34]	**1.80 ± 0.60**	**1.80 ± 0.60**
[35]	**1.60 ± 0.40 (right)** **1.50 ± 0.40 (left)**	**1.70 ± 0.40 (right)** **1.50 ± 0.40 (left)**
[36]	**3.30 ± 0.90 (right)** **3.50 ± 1.10 (left)**	**3.90 ± 1.30 (right)** **4.00 ± 1.40 (left)**
[37]	**3.90 (M)** **3.20 (F)**	**4.10 (M)** **3.50 (F)**
[38]	**3.70 ± 1.10**	
[11] *	**1.93 (thoracic right)** **1.82 (thoracic left)** **2.44 (lumbar right)** **2.42 (lumbar left)** **4.29 (sacral right)** **4.34 (sacral left)**	
[26]	**2.00**	-
[39]	Users and non-users of hormonal contraceptives: **4.20 and 5.60 (1st measurement)** **4.00 and 5.40 (2nd measurement)**	
[17]	**0.96 ± 0.15 (posterior layer)** **0.41 ± 0.05 (middle layer)**	-
[40]	**3.00 ± 0.50**	-

### 3.2. Superficial Fascia Thickness

Fascial tissue continuity, such as the connectivity among the SF and DF, paves the way to also study the SF in the LBP scenario, since alterations in the SF could have an impact on force transmission along the whole body.

The term “superficial fascia” appeared only at the end of the nineteenth century with studies on the abdomen and pelvis. In 1825, Velpau affirmed that it is present throughout the body, but unfortunately, no one systematically continued its characterization, thus creating difficulties in its identification and nomenclature [44].

Indeed, regarding SF thickness, in the literature, there is a paucity of significant works [26,27], with little to no data in the LBP scenario (due to a lack of data in terms of its correlation; see Table 3). The SF of the thoracolumbar region does not have a proper name like the deep one. It appeared as a fibroadipose structure made up of interconnected lamellae extending between the dermis and the DF [2]. Hammoudeh et al. [26] reported a higher average SF thickness in the posterior part of the thorax (0.6 mm). Abu-Hijleh et al. [27] evaluated the thickness of the membranous layer of the SF from formalin-fixed cadavers (potential bias for comparison with [26]) in different regions of the body, concluding that the arrangement and thickness varied according to body region, surface, and sex. In the posterior/back trunk, female thickness is significantly higher than the male thickness, and for females, the posterior region is thicker than the anterior abdominal wall.

**Table 3:** Thickness (mean ± standard deviation) of the SF from the literature—divided between LBP and non-LBP subgroups. Unique cell: not specified or not considered separately between LBP and non-LBP subjects. * Mean ± standard error.

Reference	Thickness (mm)	
	Non-LBP	LBP
[26]	**0.600**	-
[27] *	**0.145 ± 0.015 (M)** **0.165 ± 0.009 (F)**	

### 3.3. Subcutaneous Tissue Thickness

Subcutaneous tissues could provide more specific (local) information about fat distribution at a specific site (i.e., lumbar) than global body mass index (BMI). In the literature, different authors have investigated subcutaneous tissue thickness [4,23,26,30,32,36,37,41], even with some methodological differences in tissue localization (i.e., considering the fat component). In Table 4, the literature results on subcutaneous tissue thickness are reported, where an increase can be noted in LBP populations when compared to controls [4,23,36,37]. In addition, in some studies, LBP populations were older than controls. According to some authors [23], the mean subcutaneous fat thickness was thicker in symptomatic women than in men, while there was no significant difference in the asymptomatic groups. For example, refs. [36,37] demonstrated higher thickness in females than in males, in both LBP and non-LBP groups, where the female populations were older. The same authors [23] reported that subcutaneous tissue thickness was better than BMI in predicting symptomatic patients with LBP. For the abdomen (thus for a different region), Kanehisa et al. [45] evidenced that aging was associated with a decrement in muscle thickness and an increment in subcutaneous fat, rather than muscle thickness, which reflected waist circumference, regardless of age and sex. In terms of dysfunctions, Calvo-Lobo et al. [32] demonstrated that lumbar erector spinae contractile properties during tensiomyography assessments may be widely correlated with subcutaneous tissue thickness.

**Table 4:** Thickness (mean ± standard deviation) of subcutaneous tissue from the literature—divided between LBP and non-LBP subgroups. Unique cell: not specified or not considered separately between LBP and non-LBP subjects. Gray cells: works that include LBP and non-LBP groups for comparisons. * Data derived from Figure 3 of the cited study.

Reference	Thickness (mm)	
	Non-LBP	LBP
[4] *	**4.8 ± 0.6**	**5.3 ± 0.5**
[30]	-	**8.32 (left)**
[32]	**3.0 (median)**	-
[37]	**5.0 ± 1.9 (M)** **7.4 ± 3.3 (F)**	**5.3 ± 2.9 (M)** **11.1 ± 4.7 (F)**
[36]	**6.4 ± 3.0 (right)** **6.0 ± 2.8 (left)** **5.0 ± 1.9 (M)** **7.4 ± 3.3 (F)**	**8.6 ± 4.8 (right)** **8.4 ± 4.7 (left)** **5.3 ± 2.9 (M)** **11.1 ± 4.7 (F)**
[41]	-	**25.4 (L3–L4)** **29.1 (L4–L5)** **32.2 (L5–S1)**
[23]	**14.2 ± 8.7 (L1–L2)** **14.8 ± 8.8 (L2–L3)** **18.7 ± 10.4 (L3–L4)** **25.6 ± 12.6 (L4–L5)** **30.2 ± 13.3 (L5–S1)**	
	**10.8 ± 4.5**	**22.4 ± 9.7 (F)** **17.9 ± 9.7 (M)**
[26]	**<4.0**	-

## 4. Discussion

In recent decades, the fascial tissues have been demonstrated to play a key role in a variety of musculoskeletal dysfunctions, due to their force transmission capacity, structural properties to form special compartments (i.e., the SF around the subcutaneous vein, ensuring vessel patency [27]), and free nerve concentration that leads to a sensory organ. Therefore, fascial tissues have a fundamental role in non-specific musculoskeletal disorders (i.e., LBP). The aim of the present work was to investigate the morphology (i.e., thickness) of the soft tissues (i.e., SF, DF, and subcutaneous tissue) by collecting literature works investigating non-LBP and LBP subjects. Thickness values were reported to highlight their variability, also considering the influence of different biases (e.g., sex, age, spinal levels, and methods).

### 4.1. Answer to Research Question 1: Typical Thickness Values and Variability for SF, DF, and Subcutaneous Tissue in the Thoracolumbar Region in Asymptomatic Individuals

Table 2, Table 3 and Table 4 highlight the variability between different works and data reported. In healthy individuals, the DF showed cranio-caudal variability likely associated with the vertebral level, while the available data on SF in asymptomatic populations remain sparse, often limited to just two reported studies involving ultrasound (US) assessments, one of which was ex vivo. However, differences according to body regions have been reported by authors. The subcutaneous tissue has been extensively studied, particularly through US and MRI, showing variability along the cranio-caudal direction (caudal thicker). In general, further stratification according to factors such as age, sex, and BMI is needed to better define normal reference values.

### 4.2. Answer to Research Question 2: Thickness Variability in Relation to Factors Such as Clinical Conditions (i.e., Non-LBP vs. LBP Individuals), Age, Sex, and Surrounding Structures

In subjects with LBP, an equal or greater thickness of the TLF was found (i.e., US or ex vivo assessments); however, this result could have been influenced by biases, as in most cases, the subjects with LBP were older than the control groups. This aspect still has gaps that need deeper investigation to assess if LBP is strongly correlated with aging. Regardless, even if the sensitivity of the DF to hormonal changes was demonstrated by [39], a specific correlation between its thickness and age or sex is controversial.

Moreover, some authors have reported the impact of treatments on the change (e.g., reduction) in connective tissue thickness, but further considerations must be made on a case-by-case basis, considering both the specific treatment and the initial population (i.e., LBP or non-LBP). The paraspinal muscles (such as multifidus and erector spinae) are enveloped in fascial structures, thus becoming interesting targets in LBP investigation, as well as for statistical correlations with TLF thickness.

Regarding functional relationships, as an example, some authors [35] investigated the differences in TLF and multifidus between athletes with and without chronic LBP—also finding that chronic LBP subjects exhibited a greater disorganization of the TLF morphology in comparison to healthy athletes. Specifically, the same authors compared a cross-sectional area at resting and during muscle contraction, finding a reduced difference of multifidus cross-sectional area between the two scenarios when compared to a healthy control group. Therefore, in subjects with LBP, during muscle contraction, the tissue deformation is different compared to those without LBP, probably due to differences in the fasciae surrounding them (again, to report the continuity and influences among structures).

In the literature, there is a much larger number of studies (than for SF) regarding the thickness of the subcutaneous tissue (i.e., from US and MRI assessments). The results showed a thickening in subjects with LBP compared to controls, and in women compared to men (in LBP). In general, further research should be conducted on age- and sex-balanced cohorts to identify potential correlations with these variables and relative root causes. Furthermore, a correlation between the thickness of the subcutaneous tissue and the functionality of the muscles could be analyzed. In the literature, there are a variety of works that have discussed the quantity of paraspinal intramuscular fat infiltrations (e.g., MRI or magnetic resonance spectroscopy [46,47,48]) to analyze the implications of muscular tissue substitution with adipose tissue in case of dysfunctions. For example, Venkatesan et al. [43] affirmed that an increase in fat infiltration of paraspinal muscle, a decrease in the cross-sectional area of the lumbar multifidus, and an increase in TLF thickness characterize the degeneration of lumbar muscle in chronic LBP. Goubert et al. [49] demonstrated differences in muscle structure and muscle function between different LBP populations [41].

### 4.3. Sex and Age Differences

Although some studies have reported greater subcutaneous fat thickness in women and age-related muscle changes, the data are generally inconsistent due to unbalanced cohort designs. The DF may respond to hormonal changes [39], but its exact behavior across sex and age remains controversial.

Bhadresha et al. [41] observed that muscular content declines with age, and individuals with LBP showed more subcutaneous fat infiltration than controls. This aligns with the hypothesis that age, sex, and LBP interact in complex ways that influence the morphology of soft tissues in the thoracolumbar region.

To clarify these relationships, future studies should include age- and sex-balanced cohorts, with appropriate stratification and statistical analyses.

### 4.4. Clinical Relevance and Functional Implications

Fascial tissues, particularly the TLF, act in spinal stability and muscle function. Their thickening and disorganization in individuals with chronic LBP, as observed in athletes [35], suggest that these structural changes may contribute to dysfunctional movement patterns or persistent pain.

Specifically, reduced deformation of the multifidus during muscle contraction in LBP subjects implies altered mechanical interactions with the surrounding fascia. This further supports the concept of the fascia as a dynamic, responsive tissue, integral to musculoskeletal health.

The presence of increased subcutaneous fat, intramuscular fat infiltration, and fascial thickening in LBP cohorts could serve as potential biomarkers for dysfunction severity. Dynamic imaging tools, such as ultrasound, may enhance the clinical relevance of these markers by providing real-time insights into tissue behavior during movement or contraction.

### 4.5. Limitations and Future Developments

A limitation of this study is the fact that a meta-analysis was not conducted in terms of data correlations (thickness) with other variables such as age, BMI, sex, methods, spinal levels (only presented in a narrative form), and previous medical records. Moreover, from a methodological point of view, this review could be further extended in a systematic form and include additional reviewers for article screening and databases (i.e., Scopus).

Based on the thickness variation along the cranio-caudal direction, the differences reported in the DF could be correlated with the spinal level in future work.

This work also analyzed the SF as part of the fascia system, due to its proximity to the subcutaneous tissue and direct relationship with the DF through retinacula cutis [2]. Regarding the SF, only two results reported values from US and ex vivo assessments, reflecting the recent interest in this structure compared to others. However, a variation in terms of arrangement and thickness according to body regions, body surface area, and sex has been reported. However, this review highlights the limited knowledge currently available about this tissue.

Indeed, in addition to paraspinal muscle cross-sectional areas, intramuscular fat fraction was hypothesized to be a predictive biomarker for dysfunctions (e.g., pain and disability) and relative severity. This fat could be better investigated in relation to LBP (i.e., recurrence), aging, BMI, and TLF thickness. To investigate all of these properties and correlations, further studies could be conducted in the future, for example, through ultrasound dynamic evaluations.

Finally, a limitation of the present review is the restricted database search (PubMed and ScienceDirect), which may have led to the exclusion of relevant studies indexed in other databases such as Scopus or Embase. Future studies could expand the search strategy to enhance comprehensiveness.

## 5. Conclusions

The strength of this study is that it offers, for the first time, a comprehensive review of fascial tissue thickness (also in relation to subcutaneous tissue) in a non-LBP scenario, as well as a comparison (when data are available) with LBP groups.

The collected data show significant variability in the description of soft tissue properties and, consequently, a lack of standard references.

To conclude, morphological information of the fascia in LBP patient assessment/prevention/treatment, tissue engineering (e.g., [50]), and similar must be guided by the knowledge of their anatomical characteristics and variability, as well as the ones of the surrounding structure, in the LBP scenario. For example, in clinical practice, structures could be identified and interventions managed based on their properties, where thickness may be considered as a biomarker for musculoskeletal dysfunction through a classification approach. Indeed, the values collected in this review could be useful for the validation of automatic techniques that identify, segment, and classify these structures from bioimages. Finally, a practical implication is the possibility of targeting the treatment (e.g., physical therapy) on that specific fascial layer, and then monitoring the patient in the follow-up. In the future, a dynamic evaluation of fascial thickness can provide further insights about its deformability and, therefore, extend the knowledge to functional implications and rehabilitation. Indeed, fascial anatomy reveals a strong mechanical relationship between muscles and the deep fascia, both of which contribute to active movement and respond to passive motion. In contrast, the superficial fascia and retinacula cutis appear to be less directly involved in movement; however, their higher density of free nerve endings compared to deeper structures suggests a prominent role in peripheral sensory input, particularly in response to superficial pressure or subcutaneous alterations.

Thus, all of these findings highlight the role of fascial tissue variability as a potential hub to define corrective and preventive actions for patient- and site-specific evaluations and care.

## Data Availability

No new data were created or analyzed in this study. Data sharing is not applicable to this article.

## Abbreviations

The following abbreviations are used in this manuscript:

LBP	Low back pain
BMI	Body mass index
DF	Deep fascia
TLF	Thoracolumbar fascia
SF	Superficial fascia
MRI	Magnetic resonance imaging
US	Ultrasonography
